# Cerebrospinal fluid microglia and neurodegenerative markers in twins concordant and discordant for psychotic disorders

**DOI:** 10.1007/s00406-016-0759-5

**Published:** 2016-12-30

**Authors:** Viktoria Johansson, Joel Jakobsson, Rebecca G. Fortgang, Henrik Zetterberg, Kaj Blennow, Tyrone D. Cannon, Christina M. Hultman, Lennart Wetterberg, Mikael Landén

**Affiliations:** 1 Department of Medical Epidemiology and Biostatistics, Karolinska Institutet, Stockholm, Sweden; 2 Institute of Neuroscience and Physiology, University of Gothenburg, Gothenburg, Sweden; 3 Department of Psychology, Yale University, New Haven, USA; 4 UCL Institute of Neurology, Queen Square, London, UK; 5 Department of Psychiatry, Yale University, New Haven, USA; 6 Department of Clinical Neuroscience, Karolinska Institutet, Stockholm, Sweden

**Keywords:** Schizophrenia, Bipolar disorder, Biomarker, Cerebrospinal fluid, Twin study, Neuroinflammation

## Abstract

Schizophrenia and bipolar disorder are debilitating psychiatric disorders with partially shared symptomatology including psychotic symptoms and cognitive impairment. Aberrant levels of microglia and neurodegenerative cerebrospinal fluid (CSF) markers have previously been found in schizophrenia and bipolar disorder. We aimed to analyze familial and environmental influences on these CSF markers and their relation to psychiatric symptoms and cognitive ability. CSF was collected from 17 complete twin pairs, nine monozygotic and eight dizygotic, and from one twin sibling. Two pairs were concordant for schizophrenia, and 11 pairs discordant for schizophrenia, schizoaffective disorder or bipolar disorder, and four pairs were not affected by psychotic disorders. Markers of microglia activation [monocyte chemoattractant protein-1 (MCP-1), chitinase 3-like protein 1 (YKL-40), and soluble cluster of differentiation 14 (sCD14)], markers of β-amyloid metabolism (AβX-38, AβX-40, AβX-42 and Aβ1-42), soluble amyloid precursor proteins (sAPP-α and sAPP-β), total tau (T-tau), phosphorylated tau (P-tau), and CSF/serum albumin ratio were measured in CSF using immunoassays. Heritability of the CSF markers was estimated, and associations to psychiatric and cognitive measurements were analyzed. Heritability estimates of the microglia markers were moderate, whereas several neurodegenerative markers showed high heritability. In contrast, AβX-42, Aβ1-42, P-tau and CSF/serum albumin ratio were influenced by dominant genetic variation. Higher sCD14 levels were found in twins with schizophrenia or bipolar disorder compared to their not affected co-twins, and higher sCD14-levels were associated with psychotic symptoms. The study provides support for a significant role of sCD14 in psychotic disorders and a possible role of microglia activation in psychosis.

**Electronic supplementary material:**

The online version of this article (doi:10.1007/s00406-016-0759-5) contains supplementary material, which is available to authorized users.

## Introduction

Bipolar disorder and schizophrenia are serious psychotic disorders associated with severe symptomatology and functional impairment. The lifetime prevalence for bipolar spectrum disorders is estimated at 2.4% [1] and at 0.4% for schizophrenia [2]. Epidemiological [3] and genetic studies [4] have found evidence of overlap, blurring the line between these disorders and suggesting common underlying pathophysiological mechanisms.

Evidence from epidemiological studies [5–8] as well as postmortem and serological studies [9, 10] indicates an involvement of the immune system in schizophrenia and bipolar illness. Meta-analyses have shown that inflammatory markers such as tumor necrosis factor α (TNF-α) and C-reactive protein (CRP) are elevated in both schizophrenia and bipolar disorder [11–14]. In the 1990s, it was proposed that chronically activated macrophages produce compounds such as proinflammatory cytokines, which predisposes the brain for developing schizophrenia and mood disorders [13, 15, 16]. This hypothesis has produced a number of studies on inflammatory mechanisms in psychiatric disorders. Cluster of differentiation 14 protein (CD14) is a co-receptor of the toll-like receptor (TLR) and is involved in microglia responses of the central nervous system (CNS) [17]. Recent studies found that the soluble form of CD14 (sCD14) was higher in plasma from patients with schizophrenia [18] and bipolar disorder compared with controls [19]. Another study using blood-derived monocytes from patients with bipolar disorder and controls found higher expression of inflammatory related genes in patients compared with controls [20].

Although such studies—using peripheral blood as substrate—provide evidence for systemic inflammation, they cannot readily be extrapolated to immunological activity in the brain. To study neuroinflammation and microglial activation, cerebrospinal fluid (CSF) is a more appropriate sampling substrate as it more closely reflects the brain biochemistry [21]. Our research group recently found higher levels of monocyte chemoattractant protein-1 (MCP-1 also referred to as CCL-2), and chitinase 3-like protein 1 (YKL-40) in CSF of bipolar patients compared with controls [19].

Cognitive impairment is a core symptom of schizophrenia [22] and is also present in bipolar spectrum disorders [23, 24]. In Alzheimer’s disease—where a progressive cognitive decline occurs—the 42 amino acid form of amyloid β (Aβ-42) starts to accumulate in the brain 20–30 years before onset of symptoms [25], which is reflected by decreased CSF concentration of Aβ-42. Aβ buildup eventually triggers a neurotoxic cascade that involves hyperphosphorylation of the microtubule-stabilizing protein tau, which results in increased CSF concentrations of phosphorylated tau (P-tau), and neuroaxonal degeneration, which is reflected by increased CSF concentrations of total tau (T-tau) [26]. Lower CSF concentrations of amyloidogenic Aβ1-42 have been found in elderly patients with schizophrenia compared to controls [27]. In a postmortem study, schizophrenia patients with cognitive decline showed higher levels of senile plaques than those without cognitive decline [28], but a recent review concluded that Aβ deposition was not associated with cognitive decline in late-life schizophrenia in most studies [29]. In bipolar disorder, lower CSF levels of soluble amyloid precursor protein α (sAPP-α) and β (sAPP-β) were detected compared with controls, along with higher ratios of AβX-42 in relation to amyloid β-38 (AβX-38) and Aβ-amyloidβ-40 (AβX-40) in bipolar patients [30]. Lower plasma levels of Aβ-42 and higher Aβ40/Aβ42 ratios were found in a sample of patients with bipolar disorder or depression. The findings indicate a possible disturbance of the amyloid turnover also in bipolar disorder [31].

An important limitation with these prior studies on CSF microglia and neurodegenerative markers in bipolar disorder is the cross-sectional case–control design where it is not possible to separate genetic contributions (trait) from the effect of the disease (state). One possible solution is to study twins, which may allow disentangling genetic, shared environmental, and unique environmental influences on CSF markers. We have previously used a twin model to examine the association between microscopic structures in CSF and psychosis in twins with schizophrenia and bipolar disorder [32, 33].

In the present study, we investigated CSF markers in 17 complete twin pairs with schizophrenia or bipolar disorder and in one twin sibling. We analyzed three immune-related markers (sCD14, YKL-40, and MCP-1), six amyloid-related markers (AβX-38, AβX-40, AβX-42, Aβ1-42, sAPP-α, sAPP-β), along with T-tau and P-tau. The aim was to estimate the heritability of the CSF markers and to analyze the genetic and environmental influences of the analyzed markers in a co-twin control design, as well as to study associations between psychotic symptoms in relation to the CSF markers. We also examined the CSF markers in relationship to other personality and cognitive variables in a set of exploratory analyses.

## Methods

### Recruitment of twin subjects

The twin pairs were recruited from a nationwide study of Swedish-born, same-sex twins with schizophrenia, schizoaffective disorder, bipolar disorder, and healthy control pairs [32]. All study subjects were originally recruited through the Swedish Twin Registry. In total, 18 complete twin pairs were included but one sibling from one of the recruited pairs did not agree to undergo lumbar puncture which left us with 35 twin individuals. All 35 twins consented orally and in writing to participate and received a small remuneration for their participation. The study was approved by the Ethical Review Board, Stockholm, and was performed in compliance with the Helsinki Declaration.

### Assessment procedures

All study participants first completed screening forms of The Structured Clinical Interview for DSM-IV Axis I Disorders (SCID-I) [34] and Axis II Disorders (SCID-II) [35] and the self-assessment questionnaires Schizotypal Personality Questionnaire Brief (SPQ-B), [36], Barratt Impulsiveness Scale (BIS-11) [37], Zuckerman sensation seeking scale (ZSSS) [38] and the Temperament Evaluation of Memphis, Pisa, Paris and San Diego-autoquestionnaire (TEMPS-A) [39]. BIS-11 measures the following dimensions: attentional, motor, and nonplanning factors. TEMPS-A measures the following affective temperaments: cyclothymic, dysthymic, irritable, hyperthymic, and anxious. The subjects were then interviewed with SCID-I and SCDI-II. Psychotic symptoms were assessed by a rater through the Scale for Assessment of Negative Symptoms (SANS) [40] and the Scale for Assessment of Positive Symptoms (SAPS) [41]. The level of functioning was rated using the Global Assessment Function scale (GAF, American Psychiatric Association 1994). Subsequently the subjects were administrated the vocabulary and the block design subtests of the Wechsler Abbreviated Scale of Intelligence (WASI) and the California Verbal Learning test (CVLT). Information on socioeconomic status, smoking habits, and somatic status was collected. As the lumbar puncture was performed after the initial psychiatric assessment, a complementary psychiatric assessment was performed the same day as the lumbar puncture to update information on psychiatric status, current medication, and somatic status. All patients were in remission at the time of the lumbar puncture. Finally, the diagnoses were reviewed by two of the investigators (CMH and VJ) considering information from diagnostic assessments, medical records, and a full history of lifetime psychiatric diagnostic codes of the Swedish National Patient Register (National Board of Health and Welfare, http://www.socialstyrelsen.se) covering the period 1973–2009.

### Zygosity determination

The zygosity of the twins, MZ or DZ, was validated using a panel of 47 highly multiplexed single-nucleotide polymorphisms (SNP) [42] that provide reliable and high-quality data on a range of different DNA templates. Of the 35 twins recruited for this study, 19 individuals were MZ and 16 DZ.

### Diagnosis and antipsychotic medication

There were in total 15 probands with either schizophrenia (*n* = 6), schizoaffective disorder (*n* = 2), bipolar disorder type I (*n* = 4), or bipolar disorder type II (*n* = 3), 12 unaffected co-twins, and eight healthy twin controls. In the overall statistical analyses, we collapsed the diagnoses schizophrenia, schizoaffective disorder, bipolar disorder I and II into one category designated ‘psychotic disorder.’ In secondary analyses, we collapsed the diagnoses schizophrenia and schizoaffective disorder into one category designated ‘schizophrenia’ and bipolar disorder type 1 and 2 into one category designated ‘bipolar disorder.’ Nine out of 15 probands were prescribed antipsychotic medication of the following types: haloperidol, levomepromazine, olanzapine, perphenazine, quetiapine and risperidone.

### Collection of cerebrospinal fluid

The sampling of CSF took place during March 2008–September 2011. The same clinical neurologist performed the 35 lumbar punctures. With study participant in the sitting position, the needle was inserted in vertebral interspace L3/L4, or L4/L5. The very first drops of CSF, approximately 0.6 mL, were used for microscopic examination while the following 12 mL of CSF was collected, gently inverted to avoid gradient effects, and divided into 1.0–1.6 mL aliquots that were stored at minus 80 °C pending analysis.

### Blood sampling and BMI

Blood samples were collected before the lumbar puncture with the subjects fasting at 0800 h. High-sensitivity C-reactive protein (HS-CRP) and the white blood cell count (WBC) were measured. Height and weight were recorded on the same day as blood and CSF sampling and used for calculation of the body mass index (BMI) as a heuristic proxy for body fat of the participants.

### Analysis of blood–CSF barrier function

Albumin levels in CSF and serum were measured at the Clinical Neurochemistry Laboratory in Mölndal, Sweden, by immunonephelometry on a Beckman Immage Immunochemistry system (Beckman Instruments, BeckmanCoulter, Brea, CA, USA). The method was accredited by the Swedish Board for Accreditation and Conformity Assessment (SWEDAC). Experienced and board-certified laboratory technicians who were blinded to clinical information performed all measurements. Intra- and inter-assay coefficients of variation were below 10%. To assess the blood CSF barrier function, the ratio between albumin concentration in CSF (mg/L) and serum (g/L) was calculated.

### CSF marker analyses

The CSF concentrations of MCP-1, sAPP-α and sAPP-β, and AβX-38, AβX-40, and AβX-42 were determined using the MSD^®^ Human MCP-1 Ultra-Sensitive Kit, MSD^®^ sAPP-α/sAPP-β Multiplex Assay, and MSD ^®^ Human/Rodent (4G8) Abeta-Triplex Assay, respectively, as described by the manufacturer (Meso Scale Discovery, Gaithersburg, MD, USA). CSF concentrations of P-tau, T-tau, and Aβ1-42 were measured simultaneously by the Luminex xMAP technology using the Inno-Bia AlzBio3 kit (Innogenetics, Zwijndrecht, Belgium). The MSD-derived Aβ concentrations were derived using a detection antibody against the mid-domain of the Aβ proteins, whereas we also measured Aβ-42 using the AlzBio3 kit. This kit includes a neo-epitope-specific antibody against the first amino acids of Aβ. For this reason, we denote MSD-derived Aβ concentrations AβX-38, AβX-40 and AβX-42 and AlzBio3-derived Aβ concentrations Aβ1-42 throughout the manuscript. sCD14 and YKL-40 were determined by Human sCD14 quantikine ELISA kit and Human chitinase-3 quantikine ELISA kit, respectively (R&D systems, Inc, Minneapolis, MN). All CSF analyses were performed in one round of analyses using one batch of reagents by board-certified laboratory technicians who were blinded to clinical information.

### Statistical analyses

Demographics, disease characteristics, and pharmacological treatment are presented as percentages, means (standard deviations), or medians (max, min-scores). For correlations, Pearson’s correlation coefficients were calculated where the markers were normally distributed else Spearman’s correlation was calculated and Fishers Z-transformation was used to compute confidence intervals. For heritability estimation, we performed structural equation modeling of the observed covariance in MZ and DZ twin pairs to find maximum likelihood estimates for additive genetic effects (A), shared environmental effects (C), unique environmental effects (E), and dominant genetic effects (D). Standardized maximum likelihood estimates were squared to yield proportions of phenotypic variance accounted for by each term. We tested model fit of ACE, ADE, AE, DE, CE, and E models using the *χ* ^2^ statistic, the root-mean-square error of approximation (RMSEA), and the Akaike information criterion (AIC). The AIC is a relative measure of fit [43]. Lower values indicate a better fit, and the score penalizes additional parameters and therefore favors parsimonious models [44].

In the co-twin control analysis of the disease-discordant twin pairs, the Shapiro–Wilk test of normality of the intrapair differences showed that all markers were normally distributed. We therefore used the paired *t* test in all co-twin control analyses. Associations between CSF markers and psychometric scales and neurocognitive testing results and clinical characteristics were analyzed with a mixed linear regression model with random intercepts shared within the twin pairs with age and sex included as covariates. The CSF markers entered the model as the dependent variable (outcome) and the scales results or confounding factors as independent variables (exposure). The same model was used to test differences between probands (patients with schizophrenia, bipolar disorder, or schizoaffective disorder), unaffected co-twins and healthy controls. Also here the CSF markers entered the model as dependent (outcome) variable.

Log transformations were applied when the data were not normally distributed which included the following CSF markers: sCD14, sAPP-α, sAPP-β, tau, p-tau, and the CSF/serum albumin ratio. Given the unique nature of the study, no adjustments were made for multiple testing, as we preferred to generate potential findings of interest that could be verified in future studies instead of missing potential findings of interest as might be the case with a Bonferroni correction. All analyses were performed using the SAS 9.4 statistical software (SAS Institute Inc, USA) except for the structural equation modeling where we employed the matrix algebra program Mx [45].

## Results

We obtained CSF from in total 35 twins belonging to 17 complete twin pairs and one single twin. Eleven of the pairs were discordant for schizophrenia, schizoaffective disorder, or bipolar disorder. Two pairs were concordant for schizophrenia, and four twin pairs were control pairs. Characteristics of the twins are presented in Table 1.

**Table 1:** Characteristics of the twin cohort divided into probands, co-twins, and controls (left hand side), and monozygotic and dizygotic twins (right hand side)

	Proband *n* = 15	Co-twin *n* = 12	Control *n* = 8	Monozygotic *n* = 19	Dizygotic *n* = 16
	*N* (%) or mean ± SD	*N* (%) or mean ± SD	*N* (%) or mean ± SD	*N* (%) or mean ± SD	*N* (%) or mean ± SD
Sex, male	5 (33.3)	4 (33.3)	8 (100)	9 (47.4)	8 (50.0)
Age	52.4 ± 9.6	51.1 ± 10.6	56.5 ± 5.9	54.2 ± 10.8	51.4 ± 6.9
Zygosity, MZ	8 (53.3)	7 (58.3)	4 (50.0)		
Cohabitation^a^	2 (15.4)	5 (41.7)	3 (37.5)	5 (29.4)	5 (31.25)
Education^b^	6 (40.0)	6 (50.0)	2 (25.0)	7 (36.8)	7 (43.8)
Smoke	7 (46.7)	1 (8.3)	3 (37.5)	5 (26.3)	6 (37.5)
Snuff	5 (33.3)	1 (8.3)	0 (0)	4 (21.1)	2 (12.5)
Body mass index	28.8 ± 7.9	27.5 ± 6.0	24.5 ± 2.1	27.2 ± 5.8	27.6 ± 7.2
*Psychiatric diagnoses*					
Schizophrenia	6 (40.0)	–	–	3 (15.8)	3 (18.8)
Schizoaffective disorder	2 (13.3)	–	–	1 (5.3)	1 (6.3)
Bipolar disorder type I	4 (26.7)	–	–	3 (15.8)	1 (18.8)
Bipolar disorder type 2	3 (0.2)	–	–	1 (5.3)	2 (12.5)
Age of onset	26.8 ± 9.8 (*n* = 14)			24.1 ± 10.7 (*n* = 8)	30.3 ± 8 (*n* = 6)
GAF [median (min–max)]	55 (35–80)	75 (50–100)	75 (70–90)	70 (35–90)	70 (40–100)
*Laboratory data*					
Albumin ratio	5.8 ± 1.6	4.7 ± 1.8	7.7 ± 4.0	6.5 ± 2.9	5.1 ± 2.0
Blood leukocytes	6.6 ± 2.1	5.9 ± 1.3	6.9 ± 2.8	6.5 ± 2.2	6.4 ± 1.8
High-sensitivity CRP	1.3 ± 1.3 (*n* = 13)	3.9 ± 5.8 (*n* = 11)	7.7 ± 8.3 (*n* = 7)	4.1 ± 6.1 (*n* = 17)	3.3 ± 5.3 (*n* = 14)
CRP	6.3 ± 5.3 (*n* = 2)	6.0 ± 0 (*n* = 1)	5.2 ± 0 (*n* = 1)	8.0 ± 2.8 (*n* = 2)	3.9 ± 1.9 (*n* = 2)
*Medication*					
Lithium	4 (26.7)	0 (0)	0 (0)	2 (10.5)	2 (12.5)
Anticonvulsant	3 (20.0)	0 (0)	0 (0)	1 (5.3)	2 (12.5)
Antipsychotic	9 (60.0)	0 (0)	0 (0)	5 (26.3)	4 (25.0)
Antidepressant	5 (33.3)	3 (25.0)	2 (25.0)	5 (26.3)	5 (31.3)
Somatic medication	7 (46.7)	3 (25.0)	4 (50.0)	7 (36.8)	7 (43.8)

*MZ* monozygotic, *CRP* C-reactive protein, *SD* standard deviation

^a^Living with partner

^b^Defined as studies on University level

### Heritabilities of the CSF markers

Table 2 presents the heritability analyses of the microglia markers and the neurodegenerative proteins measured in CSF. The additive genetic effects on the microglia markers sCD14 and YKL-40 were moderate, while MCP-1 was entirely driven by environmental effects. High heritability estimates were found for sAPP-α, sAPP-β, and AβX-38, while the heritability of AβX-40 was moderate. AβX-42 and Aβ1-42 were influenced by non-additive (dominant) genetic effects. The heritability of T-tau and its phosphorylated isoform P-tau were high, with additive variation contributing to T-tau and dominant variation to P-tau in the best fitting models. CSF/serum albumin ratio, a proxy for the function of the CSF-blood barrier, was influenced by dominant genetic variation.

**Table 2:** Twin correlations and ACED estimates based on the best-fitted model according to Akaike’s information criterion (AIC)

	Monozygotic twin correlations	Dizygotic twin correlations	*χ* ^2^	*p* value	Best-fitted model	Model terms ACED-model			
	*r* (CI)	*r* (CI)				A	C	E	D
sCD14	0.96 (0.83–0.99)	0.73 (0.05–0.95)	73.8	<.001	ACE	0.33	0.33	0.33	
YKL-40	0.80 (0.30–0.96)	0.66 (−0.08–0.93)	84.6	<.001	ACE	0.35	0.31	0.35	
MCP-1	0.44 (−0.31–0.85)	0.16 (−0.61–0.78)	4.05	0.54	E				
sAPP-α	0.85 (0.43–0.97)	0.40 (−0.42–0.86)	2.07	0.56	ACE	0.86	0.00	0.14	
sAPP-β	0.75 (0.16–0.94)	0.33 (−0.48–0.84)	2.89	0.41	ACE	0.79	0.00	0.21	
AβX-38	0.88 (0.50–0.97)	0.00 (−0.70–0.70)	3.76	0.29	ACE	0.68	0.19	0.14	
AβX-40	0.87 (0.50–0.97)	0.06 (−0.68–0.73)	7.04	0.07	ACE	0.33	0.12	0.55	
AβX-42	0.62 (−0.08–0.91)	0.01 (−0.70–0.71)	3.63	0.46	DE			0.46	0.54
Aβ1-42	0.57 (−0.15–0.90)	0.19 (−0.59–0.79)	2.98	0.56	DE			0.46	0.54
T-Tau	0.91 (0.61–0.98)	0.42 (−0.41–0.87)	5.51	0.24	AE	0.77		0.23	
P-tau	0.85 (0.43–0.97)	0.36 (−0.46–0.85)	7.94	0.09	DE			0.22	0.78
CSF/serum albumin ratio	0.46 (−0.29–0.86)	−0.02 (−0.72–0.69)	3.05	0.55	DE			0.26	0.74

*CI* confidence interval

### Co-twin control analysis of CSF markers in the disease-discordant twin pairs

**Fig. 1:**
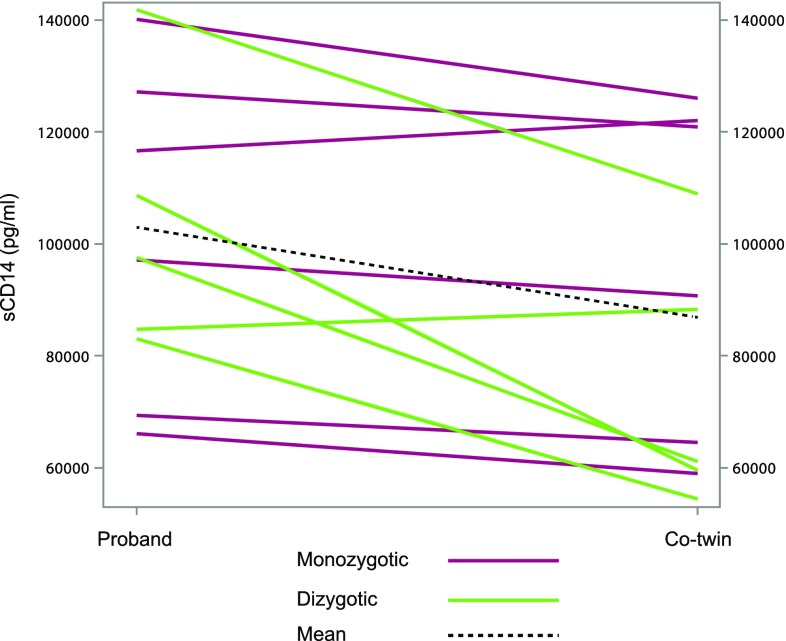
Comparison of sCD14 levels between the twin pairs where the probands are affected with schizophrenia or bipolar disorder and the co-twins are not affected and the *line* represents each pair (*n* = 11)

We continued with a co-twin control analysis of the CSF markers in twin pairs discordant for any psychotic disorder (schizophrenia, schizoaffective disorder or bipolar disorder, *n* = 11). We found significantly higher levels of sCD14 in the probands affected with a psychotic disorder compared to their non-affected co-twins, (Table 3; Fig. 1). None of the other markers showed any statistically significant difference within the pairs. We then performed a co-twin control analysis of sCD14 stratified by zygosity, psychotic diagnosis (schizophrenia/schizoaffective disorder or bipolar disorder), and by antipsychotic medication. The absolute sCD14 levels were higher in the probands compared to the co-twins in the MZ twin pairs, but the differences did not reach the significant level (Table 4). In the twin pairs with schizophrenia or schizoaffective disorder, sCD14 was significantly higher in probands compared to controls. No significant differences of sCD14-levels were seen within the pairs where the probands were medicated with antipsychotics and within the pairs where the probands did not have antipsychotics (Table 4).

**Table 3:** Results of the co-twin control analysis of the cerebrospinal fluid markers in the disease-discordant twin pairs

	Proband (*n* = 11)		Co-twin (*n* = 11)		Intrapair difference		Paired *t* test
	Mean	SD	Mean	SD	Mean	SD	*p* value
sCD14 (pg/ml)	102,930	26,431	86,843	28,607	16,086	17,847	**0.0136**
YKL-40 (pg/ml)	112,630	33,181	103,646	37,230	8984	26,791	0.2921
MCP-1 (pg/ml)	629	90.1	599	128	29.7	158	0.5468
AβX-38 (pg/ml)	1720	511	1633	473	87.2	497	0.5733
AβX-40 (pg/ml)	9889	2665	9422	2346	468	2574	0.5605
AβX-42 (pg/ml)	1117	280	988	251	129	337	0.2321
Aβ1-42 (pg/ml)	809	126	712	150	96.7	173	0.0929
sAPP-α (ng/ml)	833	380	806	270	26.8	263	0.7419
sAPP-β (ng/ml)	466	192	436	140	30.5	155	0.5286
T-tau (pg/ml)	294	89.4	299	166	−4.7	140	0.9128
P-tau (pg/ml)	38.7	10.2	38.6	18.6	0.1	14.6	0.9792

Significant results are bolded

*SD* standard deviation

**Table 4:** Co-twin control analysis of sCD14 in cerebrospinal fluid stratified by zygosity, diagnosis in the proband, and antipsychotic medication in the proband

	Proband (*n* = 11)		Co-twin (*n* = 11)		Intrapair difference		Paired *t* test
	Mean	SD	Mean	SD	Mean	SD	*p* value
*Zygosity*							
MZ *n* = 6	102,761	30,605	97,184	30,286	5578	6322	0.0831
DZ *n* = 5	103,133	23,994	74,436	23,378	28,697	19,544	**0.0304**
*Diagnosis*							
SCZ/SA, *n* = 4^a^	96,697	31,698	75,097	34,061	21,600	13,318	**0.0477**
BPD, *n* = 7^b^	106,492	24,926	93,556	25,284	12,936	20,258	0.1421
*Antipsychotics*							
Yes, *n* = 6^c^	98,023	26,623	85,112	32,426	12,911	16,972	0.1214
No, *n* = 5^d^	108,819	27,949	88,922	26,865	19,897	20,075	0.0910

Significant results marked as bolded

*SD* standard deviation, *MZ* monozygotic twin pair, *DZ* dizygotic twin pair, *SCZ* schizophrenia, *SA* schizoaffective disorder, *BPD* bipolar disorder

^a^ MZ/DZ pairs *n* = 2/*n* = 2

^b^ MZ/DZ pairs *n* = 4/*n* = 3

^c^ MZ/DZ pairs *n* = 3/*n* = 3

^d^ MZ/DZ pairs *n* = 3/*n* = 2

### Associations between psychotic symptoms and personality traits and CSF markers

We analyzed associations between the CSF markers and psychometric scales for psychosis that included SAPS, SANS, SPQ-B, and the SCID-II screen (cluster A section). Table 5 shows that significant associations were found between higher sCD14 levels and higher scores from the SANS scale and the SCID-II screening (cluster A section). Further analysis showed associations between sCD14 and the paranoid (*t* = 2.73, *p* = 0.016) and schizotypal personality traits of the SCID-II screening (*t* = 3.39, *p* = 0.0041).

**Table 5:** Results from the mixed regression analysis between the CSF markers and psychometric scales for psychotic symptoms and personality traits

	SANS				SAPS			
	Estimate	SE	*t* value	*p* value	Estimate	SE	*t* value	*p* value
MCP-1	0.10	0.86	0.12	0.91	1.05	1.80	0.58	0.57
YKL-40	256	263	0.97	0.35	401	432	0.93	0.37
sCD14	0.0021	0.0009	2.28	0.037	0.0029	0.0015	1.99	0.064
sAPP-α	−0.0009	0.0013	−0.67	0.51	−0.0031	0.0021	−1.45	0.17
sAPP-β	−0.0001	0.0013	−0.10	0.92	−0.0011	0.0021	−0.52	0.61
AβX-38	0.98	4.15	0.24	0.81	−0.65	7.18	−0.09	0.93
AβX-40	5.74	21.6	0.27	0.79	−18.58	36.91	−0.50	0.62
AβX-42	2.86	2.59	1.10	0.29	2.21	4.72	0.47	0.65
Aβ1-42	2.38	1.41	1.68	0.11	2.62	2.62	1.00	0.33
T-tau	0.0007	0.0012	0.61	0.55	0.0001	0.0020	0.07	0.95
P-tau	0.001	0.0011	1.04	0.31	0.0009	0.0018	0.49	0.63

	SPQ-B				SCID-II—cluster A			
MCP-1	4.11	3.66	1.12	0.28	1.72	3.46	0.50	0.63
YKL-40	57.6	1045	0.06	0.96	1774	884	2.01	0.063
sCD14	0.0062	0.0036	1.72	0.10	0.011	0.003	3.70	0.0021
sAPP-α	−0.0052	0.0051	−1.02	0.32	−0.0099	0.0047	−2.09	0.054
sAPP-β	−0.0014	0.0050	−0.29	0.78	−0.0063	0.0047	−1.34	0.20
AβX-38	−2.6	16.57	−0.16	0.88	−4.71	16.04	−0.29	0.77
AβX-40	−31.49	86.15	−0.37	0.72	−39.88	83.45	−0.48	0.64
AβX-42	5.78	10.67	0.54	0.60	4.55	10.28	0.44	0.66
Aβ1-42	4.93	5.936	0.83	0.41	4.56	5.69	0.80	0.43
T-tau	−0.0014	0.0047	−0.29	0.77	0.0021	0.0047	0.45	0.66
P-tau	0.0001	0.0042	0.03	0.97	0.0037	0.0041	0.90	0.38

*SE* standard error, *SANS* scale for assessment of negative symptoms, *SAPS* scale for assessment of positive symptoms, *SPQ-B* schizotypal personality questionnaire brief, *SCID-II* the structured clinical interview for DSM-IV Axis II disorders

### Associations between other psychometric scales, cognitive functions, and CSF markers

We did an overall screening of associations between the CSF markers and the following psychometric assessment scales: SCID-II screening (cluster B and C section), BIS-11, ZSSS and TEMPS-A, and the neuropsychological tests of WASI vocabulary, WASI block design, and CVLT. An association was identified between sCD14 and scores from the BIS-11 scale measuring impulsiveness, in particular with the attentional dimension of impulsiveness (Supplement Table).

### Relationship between clinical characteristics and CSF markers

Associations between the CSF markers and the following factors were analyzed: CSF/serum albumin ratio, smoking, snuff use, ongoing or previous alcohol dependence, BMI, measures of peripheral inflammation in serum (HS-CRP and WBC), and psychotropic medication (antipsychotics, lithium, antidepressants, and anticonvulsants). Associations were found between CSF/serum albumin ratio and sCD14 (*t* = 5.04, *p* < 0.0001) and CSF/serum albumin ratio and YKL-40 (*t* = 2.31, *p* = 0.03). Associations were also found between lithium use and AβX-38 (*t* = −2.54, *p* = 0.021) and AβX-40 (*t* = −2.52, *p* = 0.023); antidepressant medication and MCP-1 (*t* = 2.73, *p* = 0.015); smoking and sAPP-α (*t* = −3.13, *p* = 0.007) and sAPP-β (*t* = −3.75, *p* = 0.002); and between snuff use and sAPP-α (*t* = −3.37, *p* = 0.004), sAPP-β (*t* = −2.82, *p* = 0.012), AβX-38 (*t* = −2.64, *p* = 0.018) and AβX-40 (*t* = −2.65, *p* = 0.018).

### Exploring the association between CSF/serum albumin ratio and sCD14 and YKL-40

**Fig. 2:**
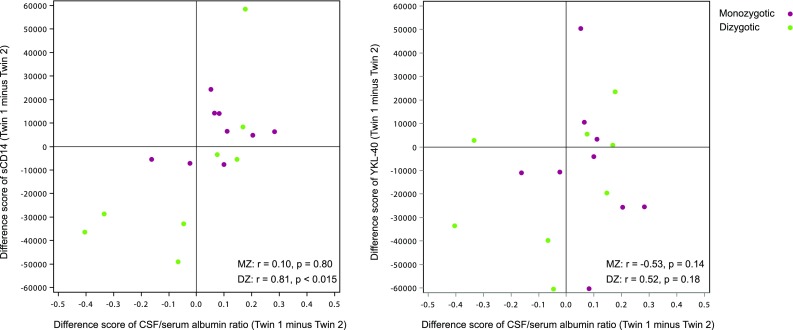
Correlations of the difference scores between the twin pairs (*n* = 17) of CSF/serum albumin ratio in relation to the CSF markers sCD14 and YKL-40

In this study and in a previous report, associations were identified between CSF/serum albumin ratio and the microglia markers sCD14 and YKL-40 [46]. The complete twin pairs (*n* = 17) were used to explore the mechanisms of these associations. In 14 out of the 17 MZ and DZ pairs, the twin in a pair with the higher sCD14 level also had the higher CSF/serum albumin ratio. Correlations of the twin pair difference scores were calculated (twin 1 minus twin 2) of CSF/serum albumin ratio and sCD14, and we found a higher correlation in the DZ pairs [*r*(8) = 0.81, *p* < 0.015] compared to the MZ pairs [*r*(9) = 0.10, *p* = 0.80], Fig. 2. The correlations of the difference sores between YKL-40 and CSF/serum albumin ratio were not significant in the DZ pairs [*r*(8) = 0.52, *p* = 0.18] nor in the MZ pairs [*r*(9) = −0.53, *p* = 0.14]. These results are not in accordance with a causal hypothesis where higher CSF/serum albumin ratio causes higher levels of sCD14 or YKL-40.

### Associations of CSF marker levels in probands, unaffected co-twins, and controls

The traditional proband-co-twin control analysis was not the most suitable statistical model due to a limited number of control pairs in our sample. As expected, given the results from the co-twin control analysis, significantly higher levels of sCD14 were found in the probands compared to the co-twins (*t* = 3.28, *p* = 0.0066).

## Discussion

Previous studies have reported aberrant levels of microglia and neurodegenerative markers in serum and CSF in patients with psychotic disorders [18, 30, 46]. Here, we estimated the heritability of 11 CSF markers in 17 twin pairs and performed a co-twin control analysis of the CSF markers in 11 pairs discordant for schizophrenia, schizoaffective disorder or bipolar disorder. In addition, we analyzed the relationship between the CSF markers and psychotic symptoms and cluster A personality traits, and finally, with an exploratory agenda, we analyzed the CSF markers in relation to additional personality traits and cognitive ability.

Our main findings include the moderate to high heritabilities of many included markers. We identified that AβX-42, Aβ1-42, P-tau, and CSF/serum albumin ratio were affected by dominant genetic variation, while MCP-1 was driven by environmental influences only in best fitting models. We also found two pieces of evidence of a possible role of sCD14 in psychotic disorder. First, higher CSF levels of sCD14 were found in the twins with psychosis compared to their unaffected co-twins. Second, associations were found between sCD14 and negative psychotic symptoms and paranoid and schizotypal personality traits. Finally, we observed a novel association between sCD14 and impulsiveness related to attention, and associations between sCD14 and borderline, antisocial, and narcissistic personality traits. The findings indicate that sCD14 partially might be involved in or reflect the disease processes of schizophrenia and bipolar disorder and that it may increase with disease severity.

No previous heritability study has been performed on the present CSF markers. In a prior report, the heritability of MCP-1 concentrations in serum was 0.63, indicating that more than 50% of the variance was due to additive genetic factors [47]. In CSF, however, we found that MCP-1 was primarily driven by unique environmental factors. This may reflect different mechanisms behind the MCP-1 expression in CNS compared to the periphery or a sample size issue. The amyloid markers, and in particular the sAPP-proteins, were influenced by additive genetics. In sAPP-α, we observed an additive genetic effect of 86%, while AβX-42 and Aβ1-42 appeared equivalently heritable due primarily to dominant genetic influences. Another interesting finding was a high additive genetic effect on T-tau, whereas its phosphorylated form, P-tau, was influenced by dominant genetic effects. The Aβ1-42 peptide and P-tau are both markers for Alzheimer’s disease. The results may indicate that interactive genetic effects could affect the regulation of the markers of Alzheimer’s disease rather than many genes with small and additive effects. However, it must be kept in mind that the sample sizes were low, and it is possible that the genetic effects attributed to dominance are actually additive genetic influences.

We found associations between higher sCD14 levels in CSF and negative psychotic symptoms, paranoid and schizotypal personality traits, and a trend association to positive psychotic symptoms. Further, we found higher levels of sCD14 in twins with schizophrenia, schizoaffective or bipolar disorder compared to their not affected co-twins. Previous studies have reported that a C to T base exchange at position 159 in the promoter region of the CD14 gene resulted in an elevated gene expression, leading to higher CD14 levels [48, 49]. Another study that investigated the genotype distribution of the CD14 C159T SNP did not find any difference between patients with schizophrenia and controls [50]. Those results are not incompatible with our finding of higher sCD14 levels in the twins with psychosis as the result in this report suggests a partial influence of the unique environment on the sCD14 levels, although we were not able to analyze the MZ pairs separately due to lack of power.

In the CNS, sCD14 is produced by activated macrophages triggered by the immune system [51, 52] and sCD14 has been suggested to be involved in microglial activation [17, 53, 54]. In addition, it has been shown previously that serum and CSF levels of sCD14 are uncorrelated, indicating that the finding of higher CSF sCD14 levels is CNS specific [19]. Antipsychotic medication may also give rise to higher sCD14 levels, but in the co-twin control analysis we could not find any difference between the twin pairs discordant for antipsychotic medication and without antipsychotics and no association between sCD14 and antipsychotic medication was found in the regression analysis. The association between higher sCD14 levels and psychosis thus provide further evidence for a role of microglia activation in psychotic disorders.

An interesting finding was the strong association between sCD14 and CSF/serum albumin ratio. Increased CSF/serum albumin ratio may indicate a disturbed function of the blood–CSF barrier, and a recent study suggests that this may contribute to increased brain levels of proinflammatory cytokines [55]. Previous studies have demonstrated higher CSF/serum albumin ratio levels in patients with schizophrenia and bipolar disorder [56, 57] as well as a correlation to negative psychotic symptoms [58]. We hypothesized that the higher sCD14 levels in CSF would be a consequence of an increased influx of proteins from serum to the CSF due to worsened integrity of the blood–CSF-barrier. Thus, we explored the association between sCD14 and CSF/serum albumin ratio by analyzing the correlation of the difference scores within the twin pairs. The result, however, was not consistent with a causal relationship between CSF/serum albumin ratio and sCD14, and despite an association the markers may be independent.

A previous study demonstrated lower CSF Aβ-42 levels in carriers of the ε4 allele of the apolipoprotein E (*APOE*) gene [59], but recent data show that this is mediated via the association of ε4 with senile plaque pathology in Alzheimer’s disease [60]. Moreover, associations have been observed between the *APOE* gene and vascular regulation in schizophrenia [61], but a recent meta-analysis did not find the *APOE* gene to be a risk factor in schizophrenia [62]. In the present study, we found no associations between the neurodegenerative markers (amyloid- and tau-proteins) and psychiatric symptoms or cognitive ability which is consistent with the view that psychosis is not a classical neurodegenerative disorder with major cell atrophy as in, e.g., Alzheimer’s disease.

Finally, we screened the results from the psychometric scales and cognitive ability against the 11 CSF markers. We found an association between sCD14 and impulsivity, in line with some previous work suggesting a relationship between immune functioning and impulsivity [63, 64]. In addition, in a study from our research group it was found that increased impulsivity may be a part of the endophenotype of patients with schizophrenia and bipolar disorder [65]. Future work examining the relationship between impulsivity and sCD14 may be of interest.

Strengths of this study include the unique collection of CSF from twins concordant and discordant for schizophrenia or bipolar disorder. The participants were thoroughly phenotyped and reexamined in conjunction to lumbar puncture. However, it is challenging to recruit twin pairs concordant and discordant for psychotic disorder volunteering for a spinal tap. The sample size of this study is therefore limited. We performed several tests and the obtained results would not withstand correction for multiple testing. In balancing the risk for type I and type II errors, we opted for presenting the results without correction for multiple testing. This means that there is a risk for false-positive findings, and results should be regarded as preliminary pending future replications in larger twin samples.

In summary, in a unique sample of twins with and without psychotic disorders, we demonstrated for the first time higher CSF sCD14 levels in twins with a diagnosis of schizophrenia or bipolar disorder compared to unaffected co-twins. This difference is interpreted as partly attributable to environmental factors such as inflammation within the CNS. In consonance with this, higher CSF sCD14 levels were associated with psychotic symptoms and paranoid and schizotypal personality traits, supporting a dimensional approach, independent of established diagnoses. Finally our study indicates moderate heritability estimates of the microglia CSF markers, high heritability estimates of T-tau, and the amyloid metabolism markers, whereas the markers of Alzheimer’s disease (AβX-42, Aβ1-42 and P-tau) were affected by dominant genetic influences. The study provides further evidence to the hypothesis of CNS inflammation in psychotic disorders and a possible significant role of sCD14.

## Electronic supplementary material

Below is the link to the electronic supplementary material.

Supplementary material 1 (DOCX 26 kb)
