# Supplementary material for: Cerebrospinal fluid microglia and neurodegenerative markers in twins concordant and discordant for psychotic disorders
Source: Eur Arch Psychiatry Clin Neurosci. 2016 Dec 30;267(5):391–402. doi: 10.1007/s00406-016-0759-5 (PMC5509775; doi:10.1007/s00406-016-0759-5)
Supplement: Supplementary file 1 — Supplementary material 1 (DOCX 26 kb) [file 406_2016_759_MOESM1_ESM.docx]

**Supplement Table**. Results from the mixed regression analysis between the CSF markers and psychometric scales and cognitive testing results.

|  | **BIS-11** | | | | **ZSSS** | | | |
| --- | --- | --- | --- | --- | --- | --- | --- | --- |
|  | **Estimate** | **SE** | **t-value** | **p-value** | **Estimate** | **SE** | **t-value** | **p-value** |
| **MCP-1** | 0.15 | 1.87 | 0.08 | 0.94 | 1.79 | 4.50 | 0.40 | 0.70 |
| **YKL-40** | 541 | 425 | 1.27 | 0.22 | 550.19 | 1184 | 0.46 | 0.65 |
| **sCD14** | 0.0032 | 0.0014 | 2.29 | 0.036 | -0.0012 | 0.0043 | -0.27 | 0.79 |
| **sAPP-α** | -0.0034 | 0.0021 | -1.59 | 0.13 | -0.0054 | 0.0060 | -0.91 | 0.37 |
| **sAPP-β** | -0.0019 | 0.0021 | -0.91 | 0.38 | -0.0058 | 0.0057 | -1.03 | 0.32 |
| **AβX-38** | -1.72 | 7.20 | -0.24 | 0.81 | -8.90 | 18.99 | -0.47 | 0.65 |
| **AβX-40** | -15.41 | 37.3 | -0.41 | 0.68 | -40.94 | 98.85 | -0.41 | 0.68 |
| **AβX-42** | 2.56 | 4.73 | 0.54 | 0.60 | -13.00 | 12.13 | -1.07 | 0.30 |
| **Aβ1-42** | 2.16 | 2.63 | 0.82 | 0.42 | -9.59 | 6.66 | -1.44 | 0.17 |
| **T-tau** | 0.0006 | 0.0020 | 0.29 | 0.78 | 0.0003 | 0.0054 | 0.05 | 0.96 |
| **P-tau** | 0.0008 | 0.0018 | 0.45 | 0.66 | -0.0003 | 0.0048 | -0.06 | 0.95 |
|  | **Temps-A** | | | | **CVLT** | | | |
|  | **Estimate** | **SE** | **t-value** | **p-value** | **Estimate** | **SE** | **t-value** | **p-value** |
| **MCP-1** | 2.11 | 2.81 | 0.75 | 0.46 | -0.0058 | 1.61 | -0.00 | 1.00 |
| **YKL-40** | 767 | 682 | 1.12 | 0.28 | -6.16 | 501 | -0.01 | 0.99 |
| **sCD14** | 0.0039 | 0.0024 | 1.63 | 0.12 | -0.0007 | 0.0019 | -0.39 | 0.70 |
| **sAPP-α** | -0.0054 | 0.0033 | -1.65 | 0.12 | 0.0047 | 0.0023 | 2.00 | 0.063 |
| **sAPP-β** | -0.0033 | 0.0033 | -1.00 | 0.33 | 0.0036 | 0.0023 | 1.56 | 0.14 |
| **AβX-38** | -1.53 | 11.15 | -0.14 | 0.89 | 6.90 | 7.64 | 0.90 | 0.38 |
| **AβX-40** | -15.07 | 57.72 | -0.26 | 0.80 | 30.5 | 40.0 | 0.76 | 0.46 |
| **AβX-42** | 1.51 | 7.36 | 0.21 | 0.84 | -0.84 | 4.93 | -0.17 | 0.87 |
| **Aβ1-42** | -0.09 | 4.09 | -0.02 | 0.98 | -1.12 | 2.76 | -0.41 | 0.69 |
| **T-tau** | 0.0015 | 0.0031 | 0.47 | 0.64 | 0.0016 | 0.0023 | 0.69 | 0.50 |
| **P-tau** | 0.0015 | 0.0028 | 0.53 | 0.61 | 0.0013 | 0.0020 | 0.64 | 0.53 |
|  | **WASI vocabulary subtest** | | | | **WASI block design subtest** | | | |
|  | **Estimate** | **SE** | **t-value** | **p-value** | **Estimate** | **SE** | **t-value** | **p-value** |
| **MCP-1** | -3.29 | 3.95 | -0.83 | 0.42 | -0.86 | 1.52 | -0.57 | 0.579 |
| **YKL-40** | 845 | 1132 | 0.75 | 0.47 | 58.4 | 482 | 0.12 | 0.91 |
| **sCD14** | -0.0020 | 0.0043 | -0.47 | 0.65 | -0.0009 | 0.0018 | -0.48 | 0.64 |
| **sAPP-α** | 0.0009 | 0.0059 | 0.15 | 0.89 | 0.0045 | 0.0023 | 1.95 | 0.07 |
| **sAPP-β** | -0.0004 | 0.0056 | -0.07 | 0.94 | 0.0046 | 0.0022 | 2.10 | 0.053 |
| **AβX-38** | -7.21 | 18.26 | -0.39 | 0.70 | 10.13 | 7.31 | 1.38 | 0.19 |
| **AβX-40** | -63.9 | 92.4 | -0.69 | 0.50 | 59.3 | 38.1 | 1.56 | 0.14 |
| **AβX-42** | -17.5 | 11.2 | -1.57 | 0.14 | 0.59 | 4.70 | 0.12 | 0.90 |
| **Aβ1-42** | -10.6 | 6.18 | -1.72 | 0.11 | -0.027 | 2.66 | -0.01 | 0.99 |
| **T-tau** | 0.0033 | 0.0055 | 0.60 | 0.56 | 0.0031 | 0.0022 | 1.45 | 0.17 |
| **P-tau** | 0.0034 | 0.0049 | 0.69 | 0.50 | 0.0023 | 0.0020 | 1.19 | 0.25 |

BIS-11= Barratt Impulsiveness Scale, ZSSS = Zuckerman sensation seeking scale, Tems-A = Temperament Evaluation of Memphis, Pisa, Paris and San Diego-autoquestionnaire, CVLT = California Verbal Learning test, WASI = Wechsler Abbreviated Scale of Intelligence.
